# Increased Mobility of Metal Oxide Nanoparticles Due to Photo and Thermal Induced Disagglomeration

**DOI:** 10.1371/journal.pone.0037363

**Published:** 2012-05-18

**Authors:** Dongxu Zhou, Samuel W. Bennett, Arturo A. Keller

**Affiliations:** Bren School of Environmental Science and Management and University of California Center for Environmental Implications of Nanotechnology, University of California Santa Barbara, Santa Barbara, California, United States of America; East Carolina University, United States of America

## Abstract

Significant advances have been made on our understanding of the fate and transport of engineered nanomaterials. One unexplored aspect of nanoparticle aggregation is how environmental stimuli such as light exposure and temperature variations affect the mobility of engineered nanoparticles. In this study, TiO_2_, ZnO, and CeO_2_ were chosen as model materials for investigating the mobility of nanoparticles under three external stimuli: heat, light and sonication. Sunlight and high power sonication were able to partially disagglomerate metal oxide clusters, but primary particles bonded by solid state necks were left intact. A cycle of temperature increase from 25°C to 65°C and then decrease back was found to disagglomerate the compact clusters in the heating phase and reagglomerate them as more open fractal structures during the cooling phase. A fractal model summing the pair-wise DLVO interactions between primary particles within two fractal agglomerates predicts weak attractions on the order of a few kT. Our study shows that common environmental stimuli such as light exposure or temperature variation can disagglomerate nanoparticle clusters and enhance their mobility in open waters. This phenomenon warrants attention since it is likely that metal oxide nanoparticles will experience these natural stimuli during their transport in the environment.

## Introduction

Recently a great amount of effort has been devoted to evaluating the potential environmental impacts of nanotechnology [1]–[4] due to the increasing applications of nanomaterials. Understanding nanoparticle aggregation in the aqueous environment is critical for assessing the fate, transport and toxicity of nanomaterials [5]. Studies conducted in different aqueous media, including synthetic matrices [6]–[16], natural waters [17], [18], and culture media [19], [20] have indicate that pH, ionic strength [7], [9]–[11], nanoparticle concentration [10], and natural organic matter [6], [8] affect nanoparticle aggregation. However, questions such as how the sintered structure and the presence of clay minerals affect the aggregation process remain to be answered.

Nanoparticles may form aggregate structures during the synthesis process. The bonding attractions between primary particles range from strong chemical bonds, established during the cooling phase of their synthesis, that form essentially unbreakable aggregates to weak physical interactions such as van der Waals forces which form agglomerates that are readily disrupted by sample preparation methods [21]–[23]. For metal oxide nanoparticles that are produced via high temperature processes, chemical bonding form during the synthesis stage when the temperature is high enough for sintering but not high enough for fast coalescence; agglomerates form at a later stage when the temperature decreases below sintering [21]. Following the suggestion of Mandzy et al. [23], we refer to particle clusters bound by irreversible chemical bonds as aggregates, and those held together by weak physical interactions as agglomerates. Once released in the environment, nanoparticles will very likely exist as agglomerated aggregates, i.e. aggregate clusters that have weaker bonds between them. This structural conformation and its effects on the stability and mobility of nanoparticles have not been addressed to any significant extent in the environmental context.

In this study, widely used metal oxide nanoparticles (TiO_2_, ZnO, and CeO_2_) were selected to study how the agglomerated aggregate structure controls nanoparticle mobility. We first probed the metal oxide agglomerate/aggregate structure via sonication and light exposure. Then the sedimentation behavior of the fractal agglomerates and aggregates was examined, followed by investigating the response to temperature variation. We report for the first time that the temperature variations can cause either agglomeration or disagglomeration of agglomerated aggregate structure depending on the heating and cooling paths. This finding is very relevant in evaluating the transport of nanoparticle transport, since it indicates that ambient temperature change, constantly occurring in open waters, can alter nanoparticle mobility. Finally a fractal aggregate model was developed to better understand the interparticle interaction between two clusters and provide an explanation for the observed phenomena, i.e. thermally-induced disagglomeration.

## Materials and Methods

### Materials

P25 TiO_2_ dry powder was obtained from Evonik Degussa (USA). CeO_2_ and ZnO dry powders were obtained from Melorium Technologies (USA). The characterization of the three metal oxides is presented in Table S2. The stock suspension was prepared by weighing the nanoparticle dry powder and suspending it in NanoPure water (NanoPure Diamond, Barnstead, MA) to achieve a 1.0 g/L concentration. The dispersion was then sonicated in a sonication bath for 30 min (Branson 2510, total power output 100 W, Danbury, CT). Chowdhury and coworkers found that 30 min in a sonication bath produced metal oxide nanoparticle suspensions with minimal hydrodynamic diameter, and further sonication led to reagglomeration of the nanoparticles [13]. The supernatant was then taken out as stock. Fresh stock suspensions were prepared daily. Samples were prepared by directly diluting the stock suspension with NanoPure water to achieve a 100 mg/L concentration. Because of the simple composition of the samples, the pH and conductivity of the samples were consistent and stable with no further pH or ionic strength adjustments (Table S2).

### Aggregate size and zeta-potential characterization

Dynamic light scattering (DLS) (Zetasizer Nano ZS-90, Malvern Instruments) was used to determine the hydrodynamic size of the metal oxide nanoparticles. A 633 nm laser source and a detection angle of 90° were used. The intensity weighted mean hydrodynamic diameter and polydispersity index (PDI) were obtained. The PDI values of all of the measurements presented in this study were below 0.4. Data was collected for 30 s for each sizing measurement. Laser Doppler Velocimetry was used to measure the electrophoretic mobility (EPM) of the nanoparticles using the Zetasizer. EPMs were converted to zeta-potential using the Smoluchowski equation [24]. Most studies were conducted at 25°C in the thermo-regulated chamber of the Zetasizer, except the set of experiments investigating the effect of heat, as described below.

### Sonication experiments

In an effort to break the agglomerates more, a 1 mL sample (100 mg/L metal oxide) was subjected to high energy sonication using a sonication probe fitted with a microtip (S-4000, Misonix Ultrasonic, USA). Samples were placed in the Zetasizer sampling chamber for sizing immediately after 2 s of sonication at 40% amplitude (input power 7 W). The input power was chosen based on preliminary results indicating that higher sonication power could not decrease the hydrodynamic size further. The 2 s sonication duration was chosen to avoid overheating, which could lead to fast aggregation and false DLS readings. Several cycles of sonication/sizing measurements were completed until no further decrease in sample hydrodynamic size was observed.

### Sedimentation experiments

Metal oxide stock suspensions (1 g/L) were observed to settle slowly over the time course of days. To quantify the sedimentation process of the agglomerated aggregates, the hydrodynamic size of 100 mg/L metal oxide samples diluted from freshly prepared stock was monitored by DLS for a week.

### Light exposure experiments

Cuvettes with 1 mL sample (100 mg/L metal oxide) were placed in sunlight for 30 min. Preliminary results showed that the bulk temperature change was less than 5°C after light exposure. Samples were subsequently placed in the Zetasizer's thermo-regulated chamber for 5 min to allow for temperature equilibration. Preliminary experiments showed that 5 min was sufficient for samples to reach the 25°C measurement temperature. Thirty 30 s sizing measurements were then carried out and the results were averaged. The experiments were carried out between 10 am to 2 pm with no cloud cover to ensure sufficient sunlight intensity.

### Temperature experiment

A cuvette with 1 mL sample (100 mg/L metal oxide) was placed in the Zetasizer chamber, and the temperature of the chamber was programmed to increase from 25°C to 65°C and then decrease from 65°C to 25°C at 1°C intervals. At each temperature, a 120 s equilibration period was allowed before size and zeta-potential data were collected in triplicate.

### TEM imaging

Transmission electron microscopy (TEM) samples were prepared by placing a drop of nanoparticle suspension on a copper grid (Ted Pella, CA) and letting it air dry overnight. Imaging was performed on a JEOL 123 microscope operated at 80 kV (JEOL, USA).

### Fractal model

Aggregates are generally fractal in nature [25], where the structure can be characterized by an exponential relationship [26],
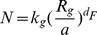
(1)where *N* is the number of primary particles in an aggregate, *k_g_* is the fractal prefactor, *R_g_* is the radius of gyration, *a* is the radius of a primary particle, and *d_F_* is the non-integer dimensionality. For an aggregate with the coordinates of each primary particle known, *R_g_* can be determined by [27],
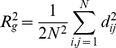
(2)where d_ij_ is the distance between primary particle i and j.

A number of studies have reported the successful application of DLVO theory [28] to evaluate colloidal stability of nanoparticle systems [14], [16], [29]–[32] by considering van der Waals attraction and electrostatic repulsion. Studies to date use DLVO calculation results between primary particles to predict the aggregation behavior of nanoparticle suspensions. Most studies have assumed that the energy-separation distance profile between primary particles can represent the interaction between aggregates [29]–[32]. However, the validity of this assumption is questionable. To evaluate this assumption we developed a 3-D MATLAB simulation code to investigate the DLVO interactions between aggregates as the agglomeration process evolves, considering a fractal configuration following Brasil, et al. [26].

**Figure 1:**
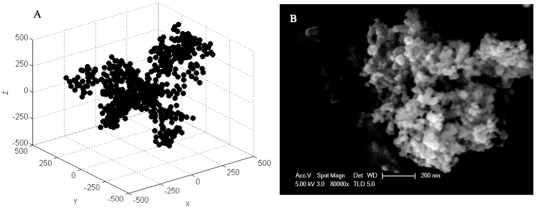
A: A representative aggregate generated by the Matlab code; B: SEM image of a TiO_2_ aggregate (Image adapted from Thio, et al. [33], with permission).

The logic in the MATLAB code is as follows. Primary particles are placed in 3D space one at a time. The first primary particle is placed at the origin (0, 0, 0). For the second and subsequent primary particles, random numbers are generated for the coordinates with the following two conditions: (a) touch at least one of the previously placed particles; and (b) not overlap with any of the existing particles. Then *R_g_* is calculated using Equation (2) and *d_F_* is calculated with Equation (1). If the calculated *d_F_* is within 5% difference of the predefined *d_F_*, the new coordinates are accepted and the code adds the next primary particle; otherwise the new coordinates are rejected, another set of coordinates is generated, and the *d_F_* evaluation is repeated. Figure 1A shows a typical fractal aggregate generated by the code, and Figure 1B a scanning electron micrograph of a typical TiO_2_ aggregate in an aqueous matrix, for comparison. After generating two fractal aggregates, the DLVO energy-separation distance profiles between them is evaluated by calculating pair-wise DLVO interactions between individual primary particles within the two fractal aggregates. The DLVO parameters for each system are presented in Table S1. The solution chemistry in the simulation can be adjusted by changing ionic strength and zeta-potential. At each condition the simulation was run 1000 times and the results were averaged. R_g_ was converted to hydrodynamic radius (R_H_) using a R_g_/R_H_ conversion ratio determined by a previous study [33] to compare simulation results with experimental data.

## Results and Discussion

### Probing the agglomerate/aggregate structure by sonication

**Figure 2:**
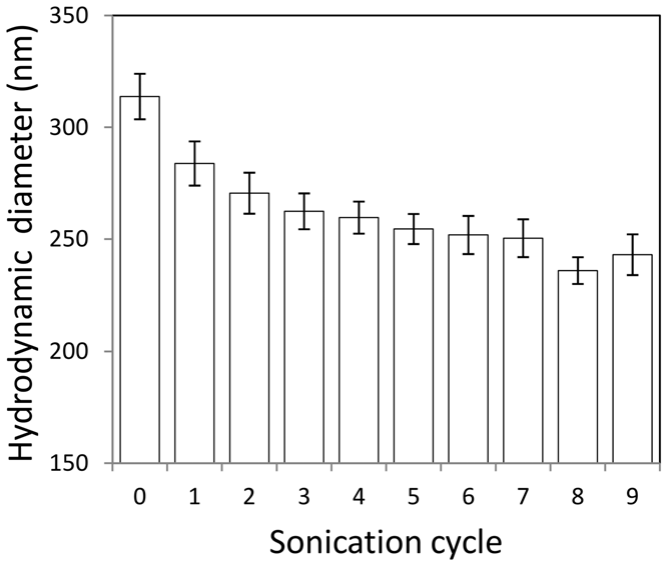
Sonication effect on particle size for a fresh stock of TiO_2_.

The nanoparticle suspension procedure using just the sonication bath for 30 min generated TiO_2_ agglomerates with hydrodynamic diameters between 250–350 nm. Several cycles of sonication/size measurement using the Misonix sonication probe were conducted until no further size decrease could be observed (Figure 2). Cavitation via sonication provides mechanical and thermal energy that promotes disagglomeration. The Misonix sonication probe (input power 7 W/mL sample volume) was unable to completely break up the agglomerates to primary particles, with the smallest observed agglomerate size as ca. 240 nm.Similar results were observed for ZnO and CeO_2_ (Figure S1).

Since DLS measurements are temperature sensitive, an increase in bulk temperature can lead to an incorrect size measurement, resulting in an artificially decreased particle size due to increased particle diffusion. Preliminary experiments with longer sonication times (5 s and 1 min) using the Misonix sonication probe heated the sample considerably leading to incorrect readings (Figure S2).

Given the high power input of the sonication probe, it is reasonable to assume that clusters without the sonication cycles were soft agglomerates of the aggregates (hydrodynamic diameter denoted as *d_s_*) while clusters after the sonication cycles were hard aggregates (hydrodynamic diameter denoted as *d_h_*). The ratio *h* = *d_s_*/*d_h_* represents the degree of agglomeration, which is 1.29 for the sample in Figure 2.

### Sedimentation

**Figure 3:**
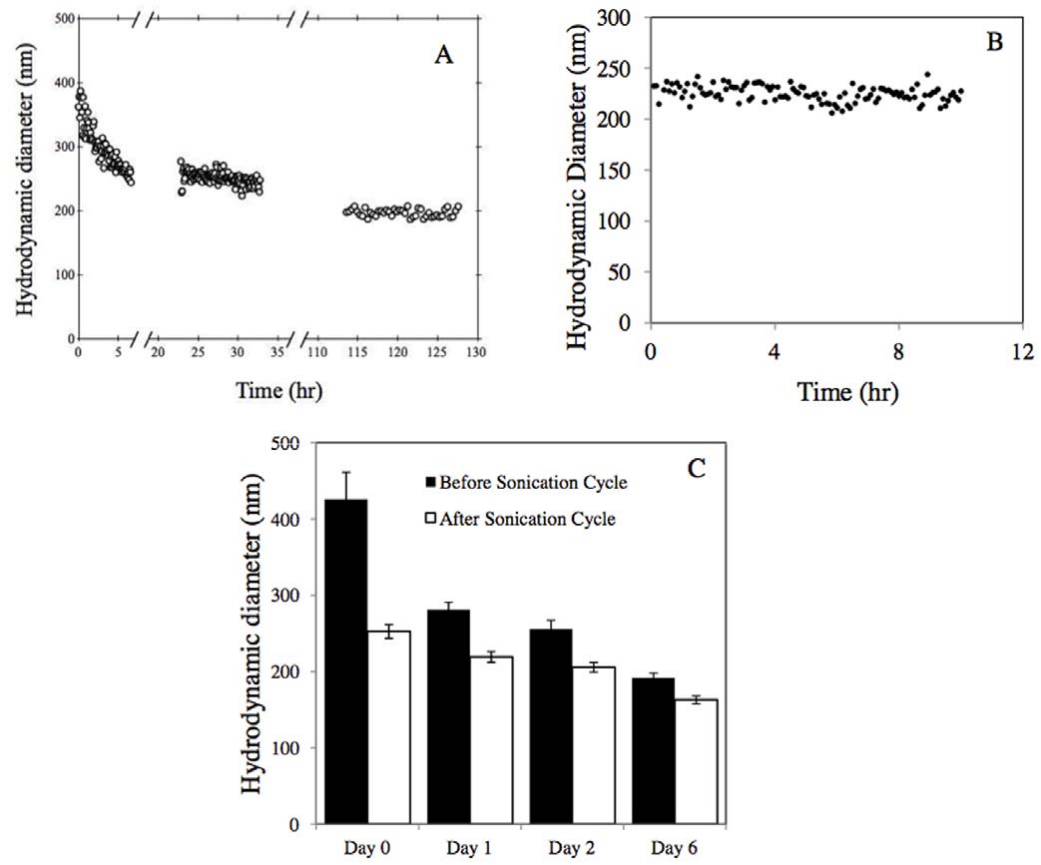
Sedimentation monitored by DLS for (A) TiO_2_ agglomerates, prepared by diluting a TiO_2_ stock suspension; (B) TiO_2_ aggregates, prepared by diluting a TiO_2_ stock suspension and sonicating with a sonication probe several times until no further reduction in hydrodynamic size was observed. (C) Hydrodynamic diameter of TiO_2_ suspension supernatant monitored for 6 days.

A significant decrease in *d_s_*, from 370 nm to 260 nm, was observed during the first 6 hr of the sedimentation experiments (Figure 3A). PDI decreased from 0.3 to 0.2. Subsequently, *d_s_* decreased at a much slower rate. Over the next 100 hr *d_s_* decreased from 250 nm to 200 nm, while PDI decreased from 0.2 to 0.1. In DLS, the hydrodynamic diameter (d_H_) is calculated assuming a single size population following a Gaussian distribution, while PDI = σ^2^/d_H_^2^, where σ is the standard deviation of the Gaussian distribution. Based on the sedimentation equation that Kajihara developed for porous particles [34], it takes roughly 10 days for a 200 nm diameter TiO_2_ agglomerate (density 4.23 g/cm^3^) to settle 1 cm (the sample depth in this study), while a 1000 nm diameter agglomerate will settle the same distance in only 20 hr (Figure S3A). A freshly prepared TiO_2_ suspension contains many large clusters, which settled down relatively quickly. As the sedimentation process continues the percentage of larger clusters decreases, therefore *d_s_* and PDI decrease. The sedimentation of a sonicated TiO_2_ sample (sonicated by sonication probe for a few cycles to break agglomerates) was also monitored by DLS for 10 hr (Figure 3B). Distinctively different from sedimentation of the freshly prepared sample, no noticeable sedimentation was observed for the sonicated sample.

**Figure 4:**
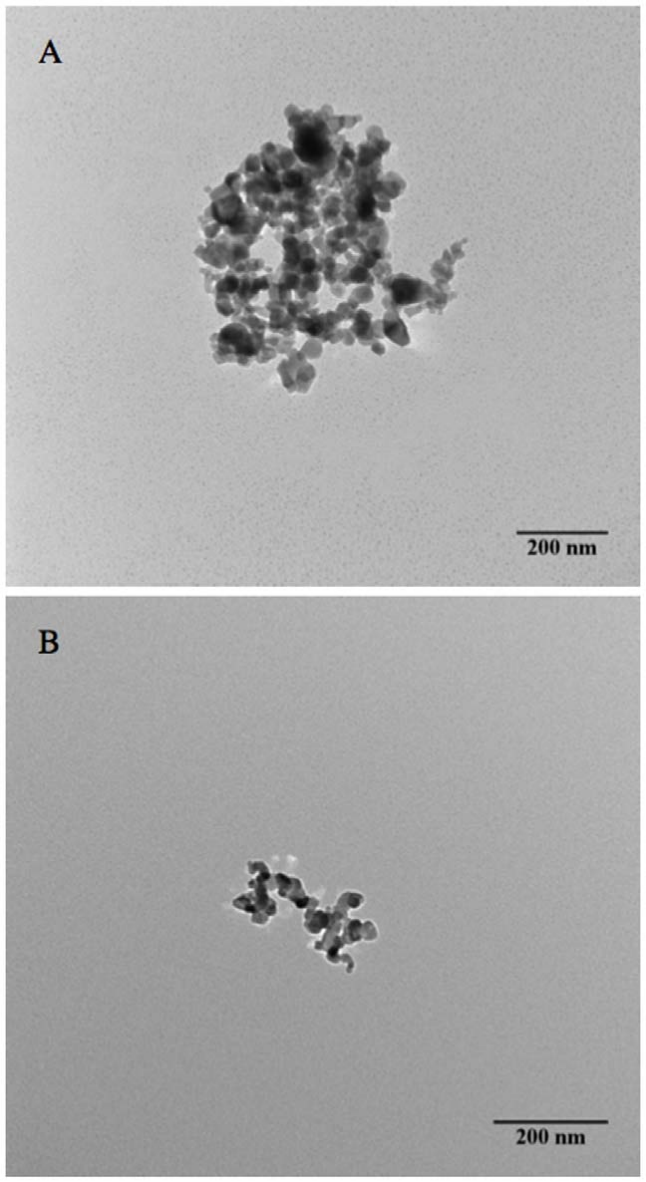
TEM micrographs of TiO_2_ agglomerate and aggregate.

Daily monitoring of the supernatant of a 1 g/L TiO_2_ stock suspension for 6 days, measuring d_z_ and PDI for sonicated and unsonicated samples, indicated that *d_s_* decreased from above 400 nm to around 200 nm, while *d_h_* decreased from 250 nm to 160 nm (Figure 3C). The degree of agglomeration, *h*, decreased from 1.68 to 1.17, indicating percentage-wise more agglomerates than aggregates settled during the course of 6 days. TEM images revealed that agglomerates form compact structures while aggregates have rather open structures (Figure 4). The sedimentation rate of fractal (porous) clusters is strongly affected by the agglomerate porosity [34]. Figure S3B shows the calculated sedimentation rate as a function of fractal dimension for clusters of equivalent hydrodynamic size (d_H_ = 200 nm). A cluster with d_F_ = 2.1 settles 3 times faster than a cluster with d_F_ = 1.5. This explains the decreased *h* during the 6 days.

### Photo-induced disaggregation

**Figure 5:**
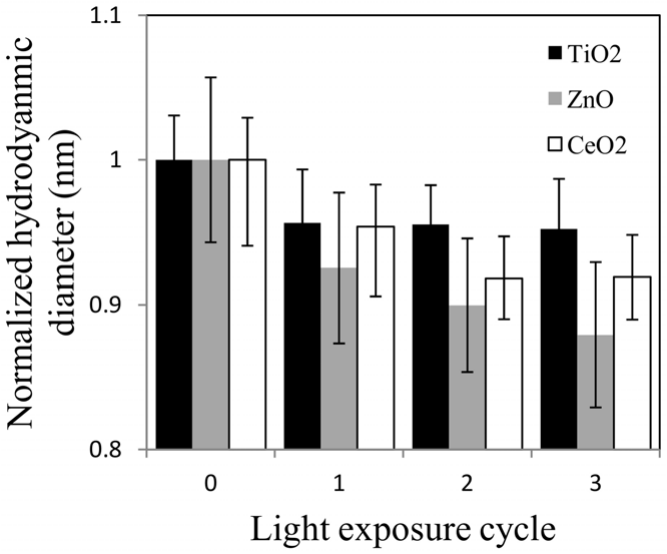
Light induced disagglomeration of metal oxide nanoparticles.

Light has been observed to disaggregate TiO_2_ nanoparticles [35]. Bennett and coworkers have shown that the hydrodynamic diameter of TiO_2_ particles was reduced significantly after the samples were subjected to either sunlight or a Xenon lamp exposure and that the particle diameters returned to their equilibrium size in the dark. Consistent behavior is observed for CeO_2_ and ZnO nanoparticles, where light can disagglomerate the dispersions (Figure 5). Similar to the sonication experiments, there appears to be an agglomerate fraction that can be disagglomerated by light, but a hard aggregate core that cannot be disaggregated remains. Localized heating of the nanoparticle agglomerates, due to exposure to natural or artificial light, provided sufficient thermokinetic energy for the clusters to disagglomerate. In this case, *h* is 1.05 for TiO_2_, 1.14 for ZnO, and 1.09 for CeO_2_.

### Effect of temperature

**Figure 6:**
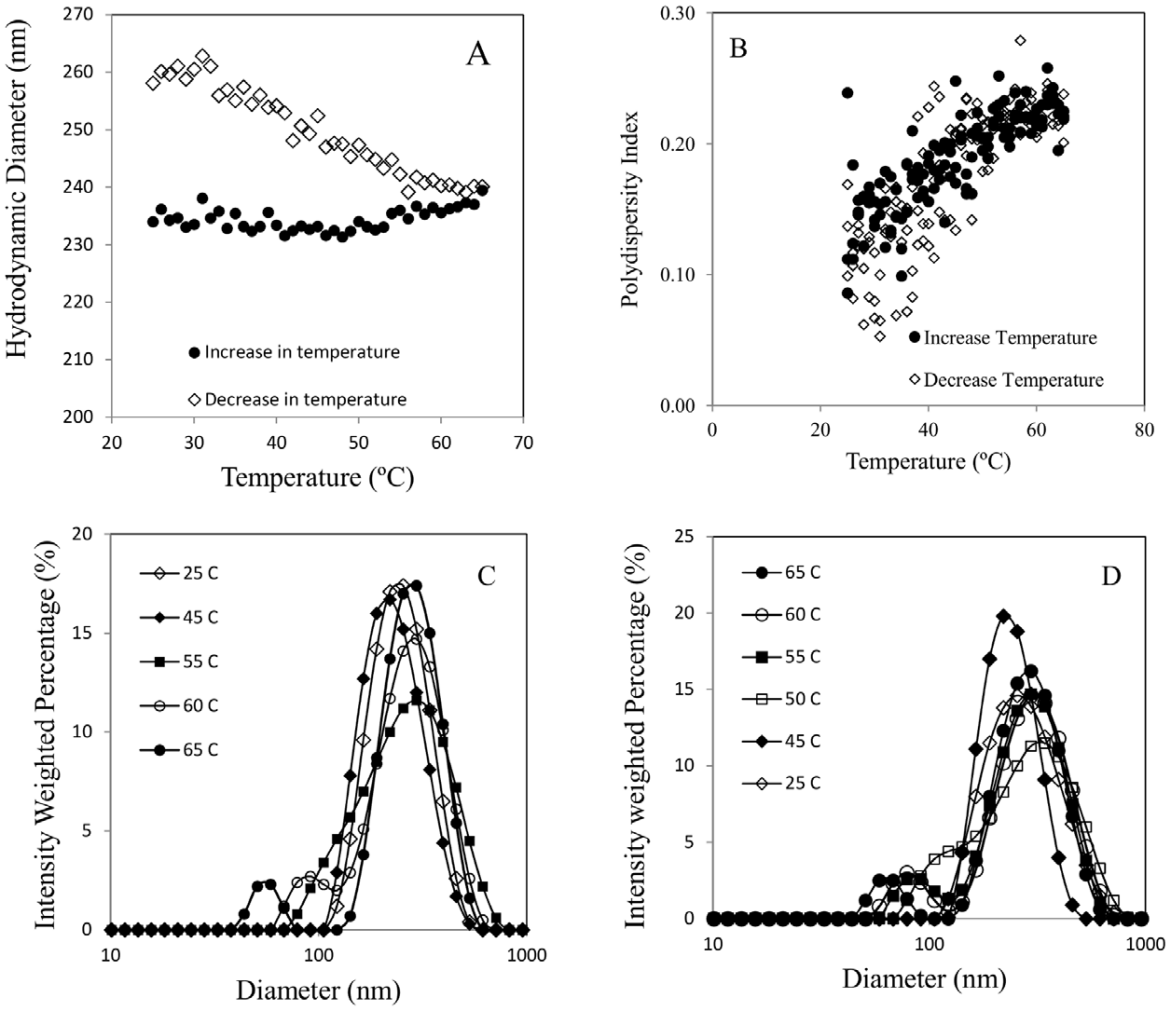
Temperature effect on: (A) hydrodynamic diameter; (B) PDI; (C) intensity weighted size distribution with increasing temperature; (D) intensity weighted size distribution with decreasing temperature.

The hydrodynamic size remained unchanged when the temperature was increased from 25°C to 65°C; however, the size increased slowly when the dispersion was cooled back to 25°C (Figure 6A). The PDI increased from about 0.1 to 0.25 when the temperature was increased, and it decreased back to 0.1 when the sample was cooled back to 25°C (Figure 6B). The change in PDI indicates a broadened size distribution at elevated temperature, which is supported by the intensity distribution data as temperature increases (Figure 6C). At 25°C, the sample was monodisperse with a peak around 255 nm. A secondary peak emerged below 100 nm once the temperature reached 55°C, and this peak became more pronounced when the sample was heated up to 65°C. During the cooling phase, the secondary peak gradually shifted towards the main peak and disappeared after the sample temperature was below 45°C (Figure 6D). Since intensity-weighted distribution is strongly biased towards larger particles (intensity is proportional to the sixth power of particle size [9]), it is very likely that TiO_2_ clusters smaller than 100 nm have emerged before the temperature reached 45–55°C, but the light scattered by the smaller TiO_2_ clusters was too weak to make a distinguishable peak. Similar disagglomeration/reagglomeration behavior was also observed for both ZnO and CeO_2_ (Figure S4).

**Figure 7:**
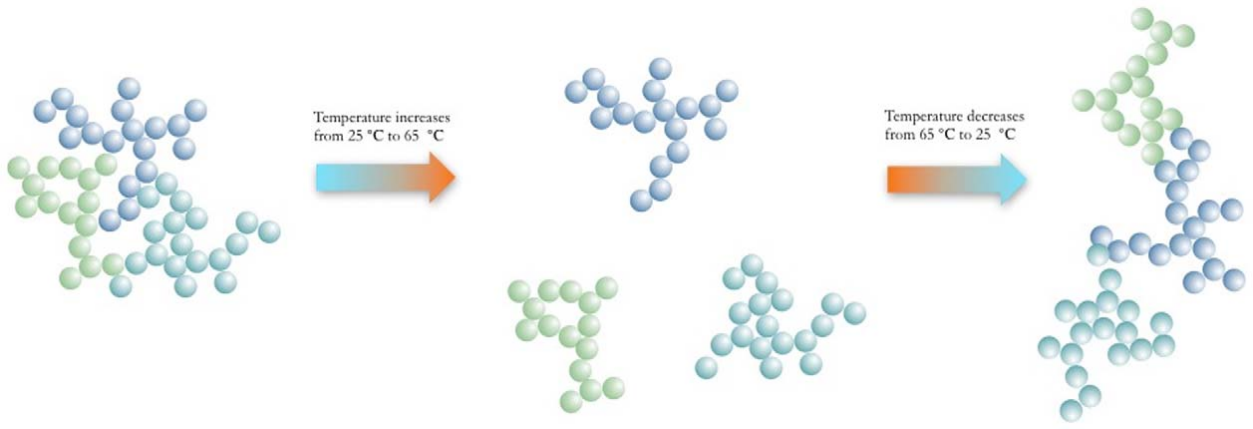
Schematic of temperature induced disagglomeration and reagglomeration.

The zeta-potential of the TiO_2_ nanoparticles decreased when the sample was heated, and increased when the temperature was reduced back to room temperature (Figure S5). A similar trend in zeta-potential with temperature was reported by another study [36]. The reduction in zeta-potential with increased temperature is not the cause of the disagglomeration, since a smaller zeta-potential would lead to less electrostatic repulsion and thus agglomeration. The higher thermal kinetic energy at elevated temperatures disrupted weak physical attractions inside the agglomerates but was unable to break the strongly bonded aggregates. The process of the disagglomeration from compact agglomerates to loose aggregates did not reduce the measured hydrodynamic size, since looser structures with the same mass yield greater hydrodynamic size. When the temperature was reduced, the weak attractions became sufficient again to hold aggregates together as agglomerates. However, the new agglomerates were not as compact as before since reagglomeration happened too fast for optimal configurations. This led to increased hydrodynamic size (Figure 6A). The schematic in Figure 7 illustrates this process.

### Interpretation via DLVO Modeling

**Figure 8:**
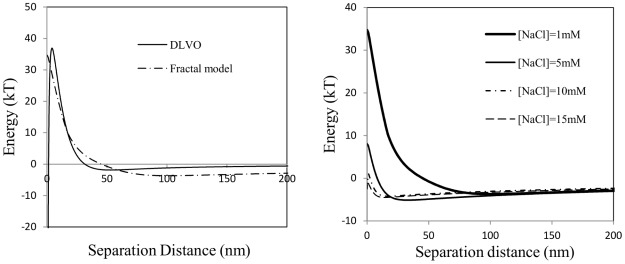
Simulation results considering (A) ionic strength = 0.1 mM; and (B) increasing ionic strength for fractal agglomerates. For particle-particle calculation, d = 200 nm. For fractal agglomerates calculation, d_primary_ = 54 nm, 20 primary particles in each aggregate, d_F_ = 1.82.

Given the complex fractal nature of the agglomerate/aggregate mixture, it is likely that the van der Waals and electrostatic interactions between TiO_2_ agglomerates/aggregate mixture deviate from those of nearly spherical particles. Figure 8A presents the interaction-separation distance profiles predicted for single particles and fractal agglomerates. The DLVO theory predicts a rather deep primary minimum (∼1000 kT), a large energy barrier (∼30 kT), and a shallow secondary minimum (∼1.8 kT) for two particles interacting. In contrast, a comparable energy barrier (∼35 kT), a shallow secondary minimum (∼3.7 kT) and essentially no primary minimum are predicted for two fractal agglomerates. This explains the soft interactions between agglomerates that can be broken by heating. It is worth noting that the simulation result is not sensitive to the d_F_ value.

With increasing ionic strength, the electrostatic repulsion is screened and the energy barrier shrinks (Figure 8B). At the critical coagulation concentration (CCC), DLVO theory predicts irreversible aggregation for the particles at the primary minimum, while for fractal agglomerates additional reversible agglomeration will occur at the slightly increased secondary minimum. To evaluate this, diffusion-limited coagulation of TiO_2_ was induced at 15 mM NaCl (CCC of TiO_2_ [37]), with the hydrodynamic diameter growing over 1000 nm. The sample was then sonicated for 2 s with 7 W using the sonication probe. Sonication completely disagglomerated the clusters back to the initial size (Figure S6), suggesting the coagulation was reversible, supporting the prediction of the fractal agglomerate model.

### Conclusion

Metal oxide nanoparticle synthesis may produce permanently sintered aggregate structures, which can further form reversible agglomerates when dispersed in aqueous media. This study shows that in open water these soft (weakly bonded) agglomerates can be disagglomerated by common environmental stimuli, such as exposure to sunlight or an increase in temperature from diurnal variations. Although not evaluated, it is likely that mechanical shocks may also result in temporary disagglomeration. The released aggregates can be much more mobile and bioavailable while the stimuli is present. Although in our experimental setting we observe reagglomeration once the stimuli are removed, in the environment it may be that the probability of interacting with another nanoparticle aggregate is much lower. Most toxicological studies are conducted at constant temperature and subdued light or under dark conditions. The effect of disagglomeration on toxicity has not been considered, or systematically evaluated. This phenomenon warrants attention since it is likely that these metal oxide nanoparticles will experience these natural stimuli during their transport in the environment.

## Supporting Information

Figure S1 Sonication effect on ZnO (A) and CeO_2_ (B) nanoparticle dispersion. Each sonication cycle lasted for 2 s with power input 7 W.
(TIF)

Figure S2 Incorrect size measurement of TiO_2_ samples due to sonication induced temperature variation. Sonication duration = 1 min. Shaded area indicates incorrect measurements.
(TIF)

Figure S3 (A) Calculated sedimentation rate of TiO_2_ as a function of hydrodynamic size, d_F_ = 2.1; (B) calculated sedimentation rate of TiO_2_ as a function of fractal dimension, hydrodynamic diameter = 200 nm. Equation adapted from Kajihara, 1971.^1^
(TIF)

Figure S4 Temperature effect on ZnO (A) and CeO_2_(B) hydrodynamic size.
(TIF)

Figure S5 Effect of temperature on the zeta-potential of TiO_2_.
(TIF)

Figure S6 Reversibility of the diffusion-limited coagulation of TiO_2._agglomerates. At every point where size decreased back to below 400 nm, sample had been sonicated by sonication probe at 7 W for 2 s. Size grows again due to the high ionic strength.
(TIF)

Table S1 Parameters used in DLVO simulations.
(DOC)

Table S2 Characterization of metal oxide Nanoparticles.
(DOC)
