# Supplementary figures and images for: Increased Mobility of Metal Oxide Nanoparticles Due to Photo and Thermal Induced Disagglomeration
Source: PLoS One. 2012 May 18;7(5):e37363. doi: 10.1371/journal.pone.0037363 (PMC3356249; doi:10.1371/journal.pone.0037363)

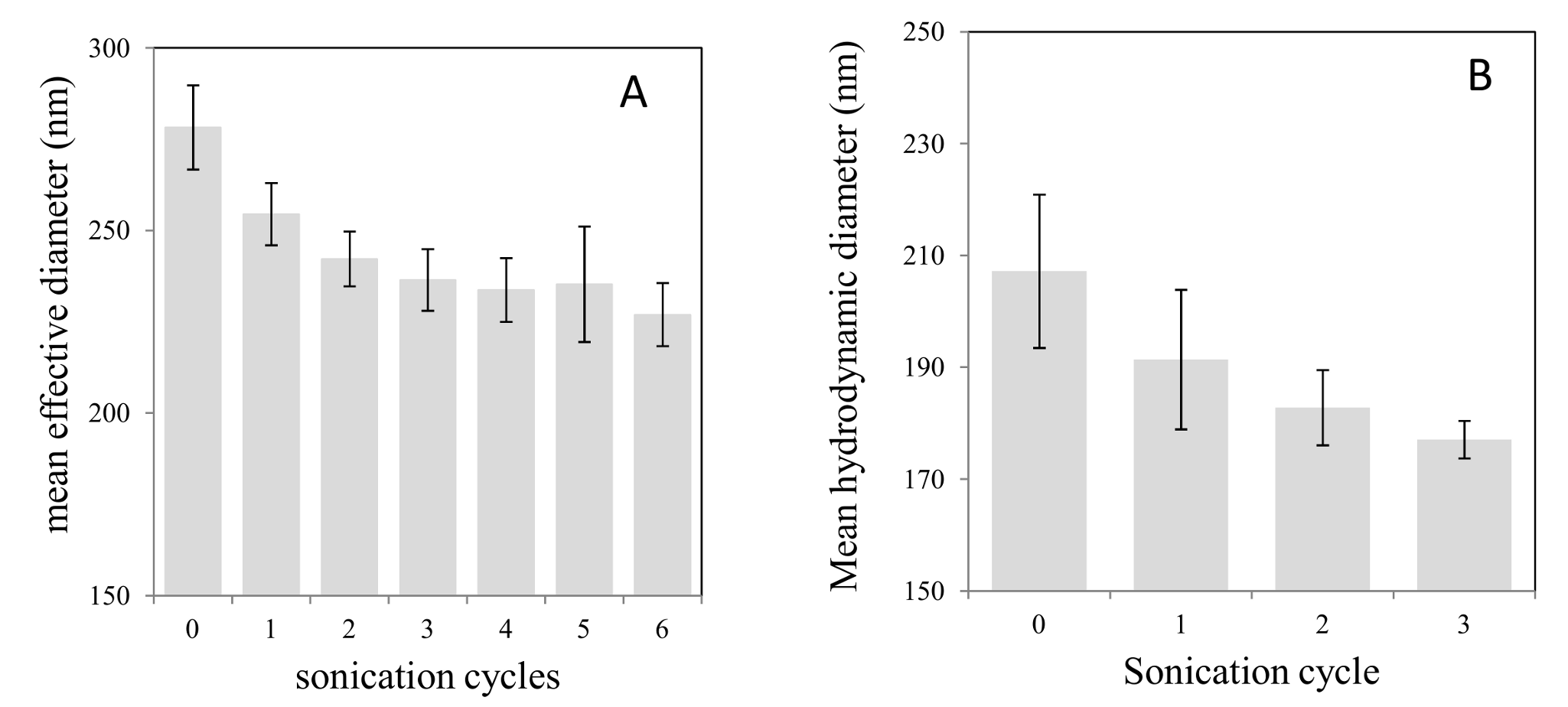

Supplement: Figure S1 — Sonication effect on ZnO (A) and CeO2 (B) nanoparticle dispersion. Each sonication cycle lasted for 2 s with power input 7 W. (TIF) [file pone.0037363.s001.tif]

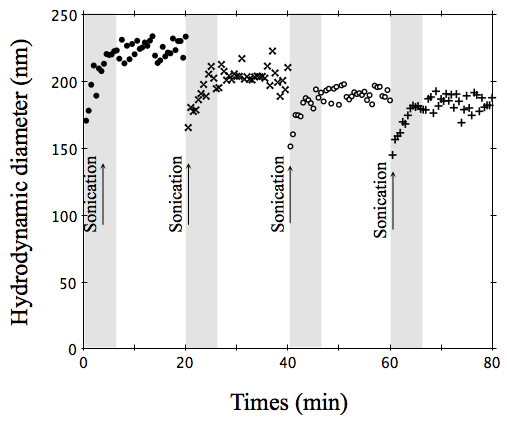

Supplement: Figure S2 — Incorrect size measurement of TiO2 samples due to sonication induced temperature variation. Sonication duration = 1 min. Shaded area indicates incorrect measurements. (TIF) [file pone.0037363.s002.tif]

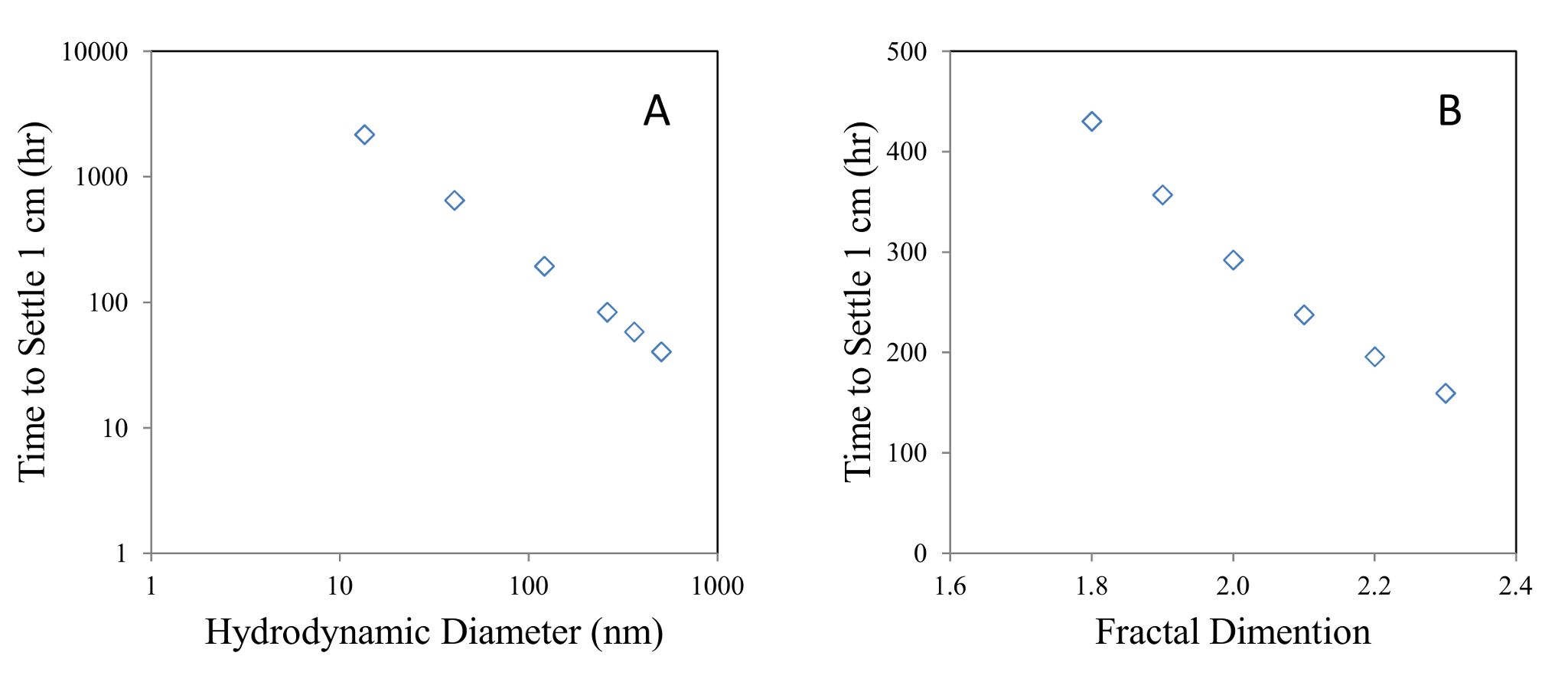

Supplement: Figure S3 — (A) Calculated sedimentation rate of TiO2 as a function of hydrodynamic size, dF = 2.1; (B) calculated sedimentation rate of TiO2 as a function of fractal dimension, hydrodynamic diameter = 200 nm. Equation adapted from Kajihara, 1971.1 (TIF) [file pone.0037363.s003.tif]

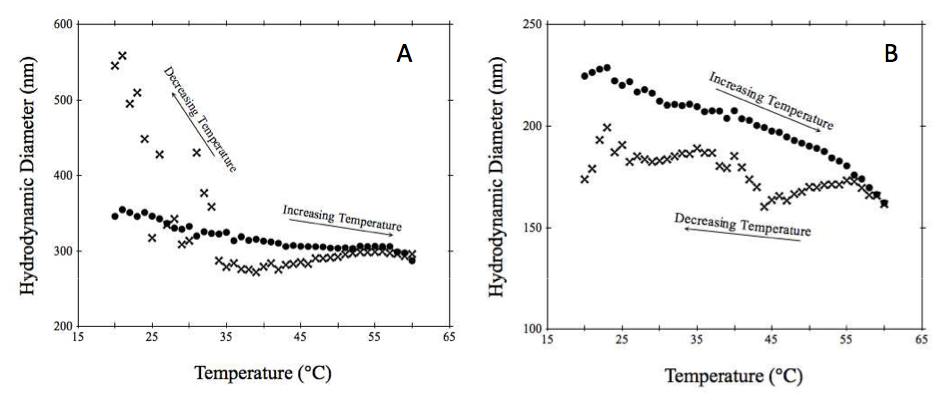

Supplement: Figure S4 — Temperature effect on ZnO (A) and CeO2(B) hydrodynamic size. (TIF) [file pone.0037363.s004.tif]

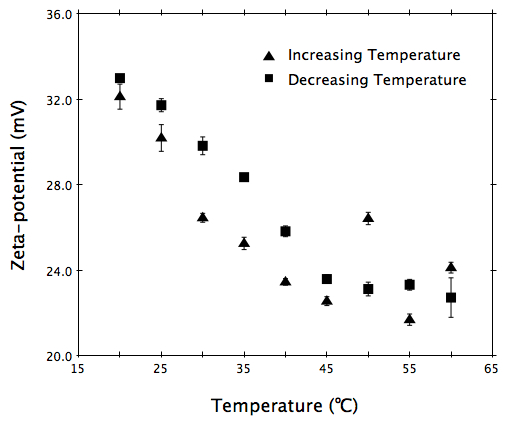

Supplement: Figure S5 — Effect of temperature on the zeta-potential of TiO2. (TIF) [file pone.0037363.s005.tif]

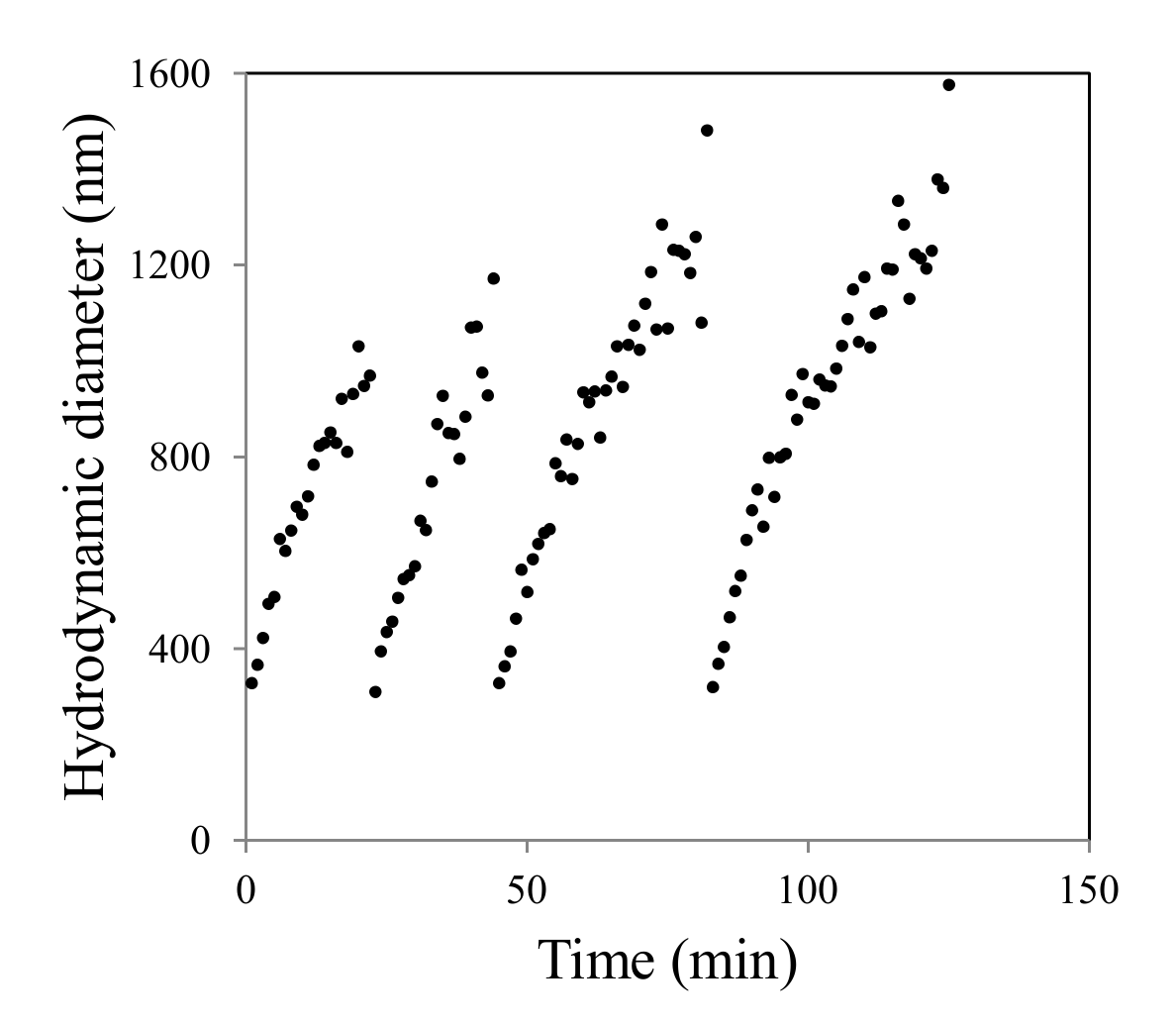

Supplement: Figure S6 — Reversibility of the diffusion-limited coagulation of TiO2.agglomerates. At every point where size decreased back to below 400 nm, sample had been sonicated by sonication probe at 7 W for 2 s. Size grows again due to the high ionic strength. (TIF) [file pone.0037363.s006.tif]
