# Supplementary material for: Increased Mobility of Metal Oxide Nanoparticles Due to Photo and Thermal Induced Disagglomeration
Source: PLoS One. 2012 May 18;7(5):e37363. doi: 10.1371/journal.pone.0037363 (PMC3356249; doi:10.1371/journal.pone.0037363)
Supplement: Table S1 — Parameters used in DLVO simulations. (DOC) [file pone.0037363.s007.doc]

Table S1. Parameters used in DLVO simulations

| **Parameters** | **TiO_2_** |
| --- | --- |
| Hamaker Constant (J)^1^ | 9.10×10^-20^ |
| Zeta-potential (mV) | 30.97 |
| Primary particle radius (nm) | 27.0 |
| Ionic strength (mM) | 1.0 |
| Temperature (ºC) | 25.0 |
| Fractal dimension (d_F_)^2^ | 1.82 |
| Fractal prefactor^3^ | 1.27 |

**References**

1. Zhang, Y.; Chen, Y.; Westerhoff, P.; Crittenden, J. Impact of natural organic matter and divalent cations on the stability of aqueous nanoparticles. Water Res. **2009**, 43, 4249–4257.
2. Karimian, H., and Babaluo, A.A. Halos mechanism in stabilizing of colloidal suspensions: nanoparticle weight fraction and pH effects. J. Eur. Ceram. Soc. **2007**, 27, 19-25.
3. Brasil, A.; Farias, T.; Carvalho, M.; Koylu, U. Numerical characterization of the morphology of aggregated particles. J. Aerosol Sci. **2001**, 32, 489–508.
