# Supplementary material for: Increased Mobility of Metal Oxide Nanoparticles Due to Photo and Thermal Induced Disagglomeration
Source: PLoS One. 2012 May 18;7(5):e37363. doi: 10.1371/journal.pone.0037363 (PMC3356249; doi:10.1371/journal.pone.0037363)
Supplement: Table S2 — Characterization of metal oxide Nanoparticles. (DOC) [file pone.0037363.s008.doc]

Table S2. Characterization of metal oxide Nanoparticles

| **Properties** | **Technique** | **unit** | **TiO_2_ Evonik** | **ZnO Meliorum** | **CeO_2_ Meliorum** |
| --- | --- | --- | --- | --- | --- |
| primary size | TEM^a^ | nm | 27 ± 4 | 24 ± 3 | rods: (67 ± 8) × (8 ± 1) (≤10% polyhedra: 8 ± 1 nm) |
| particle size in DI water | DLS^a^ | nm | 194 ± 7 | 205 ± 14 | 231 ± 16 |
| phase and structure | XRD^a^ |  | 82% anatase and 18% rutile | 100% zincite hexagonal | 100% ceria cubic |
| shape/morphology | TEM^a^ |  | semispherical | spheroid | rods (≤10% polyhedra) |
| surface area | BET^b^ | m^2^g^-1^ | 51.5 | 42.1 | 93.8 |
| IEP | zetaPALS^a^ |  | 6.2 | 9.2 | 7.5 |
| EPM in 1mM KCl | zetaPALSa | 10^-8^m^2^V^-1^s^-1^ | 2.37 ± 0.06 | 1.83 ± 0.11 | 2.19 ± 0.04 |
| purity | TGA^a^ | wt.% | 98.03 | 97.27 | 95.14 |
| moisture content | TGA^a^ | wt.% | 1.97 | 1.61 | 4.01 |
| pH of suspension | pH meter |  | 6.3 | 6.6 | 6.5 |
| conductivity of suspension | Digital conductivity meter | μS | 1.86±0.11 | 3.66±0.07 | 1.67±0.01 |
|  |  |  |  |  |  |
| ^a^Transmission electron microscopy (TEM), dynamic light scattering (DLS), X-ray powder diffraction (XRD), isoelectric point (IEP), electrophoretic mobility (EPM), and thermogravimetric analysis (TGA) were done by the UC-CEIN at UCLA. ^b^Brunauer-Emmett-Teller analysis (BET) was conducted by Dr. Ponisseril Somasundaran's lab at Columbia University | | | | | |
